# Risk of Discharge to Lower-Quality Nursing Homes Among Hospitalized Older Adults With Alzheimer Disease and Related Dementias

**DOI:** 10.1001/jamanetworkopen.2022.55134

**Published:** 2023-02-08

**Authors:** Cyrus M. Kosar, Vincent Mor, Rachel M. Werner, Momotazur Rahman

**Affiliations:** 1 Department of Health Services, Policy & Practice, Brown University School of Public Health, Providence, Rhode Island; 2 Center for Gerontology and Healthcare Research, Brown University School of Public Health, Providence, Rhode Island; 3 Center of Innovation in Long-Term Services and Supports, Veterans Administration Medical Center, Providence, Rhode Island; 4 Department of Medicine, Perelman School of Medicine, University of Pennsylvania, Philadelphia; 5 Leonard Davis Institute of Health Economics, University of Pennsylvania, Philadelphia; 6 Center for Health Equity Research and Promotion, Corporal Michael J. Crescenz VA Medical Center, Philadelphia, Pennsylvania

## Abstract

**Importance:**

Individuals with Alzheimer disease and related dementias (ADRD) frequently require skilled nursing facility (SNF) care following hospitalization. Despite lower SNF incentives to care for the ADRD population, knowledge on how the quality of SNF care differs for those with vs without ADRD is limited.

**Objective:**

To examine whether persons with ADRD are systematically admitted to lower-quality SNFs.

**Design, Setting, and Participants:**

Cross-sectional analysis of Medicare beneficiaries hospitalized between January 1, 2017, and December 31, 2019, was conducted. Data analysis was performed from January 15 to May 30, 2022. Participants were discharged to a Medicare-certified SNF from a general acute hospital. Patients younger than 65 years, enrolled in Medicare Advantage, and with prior SNF or long-term nursing home use within 1 year of hospitalization were excluded.

**Exposures:**

The quality level of all SNFs available at the patient’s discharge, measured using publicly reported 5-star staffing ratings. The 5-star ratings were grouped into 3 levels (1-2 stars [reference category, low-quality], 3 stars [average-quality], and 4-5 stars [high-quality]).

**Main Outcomes and Measures:**

The outcome was the SNF a patient entered among the possible SNF destinations available at discharge. Differences in the association between SNF quality and SNF entry for patients with and without ADRD were assessed using a conditional logit model, which simultaneously controls for differences in discharging hospital, residential neighborhood, and the other characteristics (eg, postacute care specialization) of all SNFs available at discharge.

**Results:**

The sample included 2 619 464 patients (mean [SD] age, 81.3 [8.6] years; 61% women; 87% were White; 8% were Black; 22% with ADRD). The probability of discharge to higher quality SNFs was lower for patients with ADRD. If the star rating of an SNF was high instead of low, the log-odds of being discharged to it increased by 0.31 for patients with ADRD and by 0.47 for those without ADRD (difference, −0.16; *P* < .001). The weaker association between quality and entry for patients with ADRD indicates that they are less likely to be discharged to high-quality SNFs.

**Conclusions and Relevance:**

The findings of this study suggest that patients with ADRD are more likely to be discharged to lower-quality SNFs. Targeted reforms, such as ADRD-specific compensation adjustments, may be needed to improve access to better SNFs for patients with ADRD.

## Key Points

**Question:**

Are hospitalized older adults with Alzheimer disease and related dementias (ADRD) more likely to be discharged to lower-quality skilled nursing facilities (SNFs)?

**Findings:**

In this cross-sectional analysis of more than 2 million Medicare beneficiaries hospitalized between 2017 and 2019, persons with ADRD were more likely to be discharged to lower-quality SNFs after accounting for discharging hospital, residential neighborhood, and other characteristics (eg, postacute care specialization) of all SNFs available at discharge. Results were consistent in analyses stratified by race and ethnicity, payer source, and primary diagnosis.

**Meaning:**

Improving incentives for providing postacute care to persons with ADRD may be necessary to increase access to better SNF care for persons with ADRD.

## Teaser

This cross-sectional study investigates whether hospitalized older adults with Alzheimer disease and related dementias are more likely to be discharged to lower-quality skilled nursing facilities following hospitalization.

## Introduction

As of 2019, approximately 5.8 million individuals in the US were living with some form of Alzheimer disease and related dementias (ADRD).^1^ Nursing homes are a common site of care for persons with ADRD, not only for long-term care but also postacute skilled nursing facility (SNF) care.^1,2,3,4^ Among the 1.2 to 1.4 million long-stay residents in the US, at least two-thirds are estimated to have ADRD.^2^ Among new nursing home admissions, which are commonly precipitated by hospitalization, more than 40% of the patients are estimated to have ADRD or cognitive impairment.^3,4,5^ Despite the high rate of ADRD in the nursing home setting overall and among postacute admissions, limited attention has been given to how the quality of nursing home care varies among individuals with and without ADRD.

Nursing home quality has largely improved over the past several decades.^6,7^ For several reasons, however, quality gains may not extend to persons with ADRD to the same extent as for other patients in nursing homes. For example, providing high-quality care to individuals with ADRD is costly to nursing homes, as individuals with ADRD typically require more staff time to address behavioral needs and may be more medically complex.^8^ Yet Medicare and Medicaid, the 2 largest payers for nursing home services, generally do not provide higher payments for caring for individuals with ADRD and current reimbursement rates may not adequately incentivize high-quality facilities. In addition, nursing homes with greater postacute care specialization, which are generally better resourced, may avoid treating persons with ADRD who are less likely to return home following rehabilitation.^9^ Moreover, initiatives to improve nursing home quality may exacerbate disparities between individuals with and without ADRD.^10^ The expansion of online facility report cards, for example, is unlikely to directly empower persons with ADRD, who have less capacity for making informed decisions about their nursing home care.^11^

Persons with ADRD entering lower-quality nursing homes may be more likely to experience adverse outcomes since they are already a high-risk population. Accordingly, the purpose of our study was to evaluate whether patients with ADRD discharged from the hospital are systematically admitted to lower-quality nursing homes. Although nursing home quality is multifaceted, we examine a measure of staffing quality because staffing levels have consistently been linked to better outcomes for patients in nursing homes.^12,13,14,15^ Our analyses focus on postacute patients because hospitalization frequently precipitates nursing home admission and therefore is the point when the decisions about which facility a patient enters is determined.^4,5^

## Methods

This study was approved by the Brown University Institutional Review Board and received a waiver of informed consent due to the use of deidentified claims data. Reporting followed the Strengthening the Reporting of Observational Studies in Epidemiology (STROBE) reporting guideline for cross-sectional studies.

### Data Sources

This study relied on individual-, health care facility–, and community-level data sources. Individual-level data were obtained from the Medicare Beneficiary Summary File (Medicare enrollment data), the Medicare Provider and Analysis Review (MedPAR) claims files, and Minimum Data Set nursing home resident assessments. Facility-level data were obtained from the Certification and Survey Provider Enhanced Reports database (nursing home organizational characteristics), Medicare’s Care Compare website (nursing home quality), and American Hospital Association annual surveys (hospital characteristics). The Social Deprivation Index (SDI), a composite zip code tabulation area measure of economic disadvantage, was used to characterize patients’ residences.^16^ The SDI ranges from 1 (least disadvantaged area) to 100 (most disadvantaged area). All data sources are listed and described in detail in eTable 1 in Supplement 1.

### Sample

We combined enrollment data with hospital and SNF claims to identify hospitalized traditional Medicare beneficiaries who were discharged directly to an SNF from January 1, 2017, through December 31, 2019. We applied some exclusion criteria. First, we excluded Medicare Advantage enrollees because MedPAR does not include SNF claims for these individuals. Second, to reduce heterogeneity, we excluded individuals younger than 65 years. Third, we excluded those with a nursing home stay within 1 year of their index hospitalization, identified through both claims and Minimum Data Set data. This exclusion serves multiple purposes: it removes long-stay nursing home residents who are frequently cycled in and out of hospitals,^17,18^ it ensures patients were treated under a new SNF benefit period, and it removes individuals who previously selected an SNF for postacute care, which is important for analyses of discharge location.

### Dementia Diagnosis

We classified patients as having ADRD based on the presence of an *International Statistical Classification of Diseases and Related Health Problems, 10th Revision* (*ICD-10*) code indicating dementia in any of the 25 discharge diagnosis fields of the hospital claim. With this approach, ADRD status is assigned prior to SNF admission, which is important for analyzing disparities in future discharge location. The *ICD-10* codes for ADRD were obtained from Medicare’s Chronic Condition Warehouse algorithm.^19^

### SNF Quality

The quality of SNFs was approximated with existing 5-star ratings reported on Care Compare. Although there is a variety of quality measures available on Care Compare, star ratings are the most visible to patients, families, and discharge planners, and thus are well suited for analyses of discharge location. The SNFs are assigned ratings on 3 primary domains: staffing, health inspections, and quality measures (eg, past performance on patient outcomes), which are summarized into an overall rating. We focused on the staffing rating for 3 reasons. First, staffing has been consistently linked to better outcomes for patients in nursing homes.^12,13,14,15^ Second, while quality of care is multifaceted and intangible in many ways, it is difficult to imagine the provision of high-quality care without a sufficient number of trained clinical staff. Third, the factors determinative of the staffing star rating, which today include direct care staffing levels and turnover rates, may be more easily modifiable or regulated compared with the factors determinative of the other star ratings. To facilitate interpretation, we grouped SNFs by 3 categories of staffing star rating: 1 to 2 stars (reference group: low quality), 3 stars (medium quality), and 4 to 5 stars (high quality).

### Statistical Analysis

Data analysis was conducted from January 15 to May 30, 2022. We first show the average characteristics of patients with and without ADRD including the quality level and other attributes of their destination SNFs. Patients are known to prefer SNFs nearer to their residences, and neighborhood income levels are known to be associated with SNF quality.^9,20,21,22^ Because we wanted to examine the association between ADRD and SNF quality independent of neighborhood, we graphically analyzed this association according to SDI level using local polynomial regression.

We then formally tested for differences in the likelihood of entering higher quality SNFs between patients with and without ADRD using a conditional logit (discrete choice) model.^23,24^ This approach has the advantage of being able to simultaneously account for the numerous competing characteristics of all available SNFs that a patient is choosing between. The real discharge process involves consideration of more than 1 SNF, the proximity of these SNFs, quality, and other SNF characteristics. Some SNF characteristics may be strongly correlated (eg, location and quality) or matter more depending on ADRD status. For example, patients with ADRD or their proxies may find an SNF with a dementia special care unit to be more valuable than an SNF with a higher star rating. Not accounting for these issues may mask larger differences in SNF quality by ADRD status. The conditional logit approach, however, models the observable attributes of all competing SNFs simultaneously.

In our analysis the outcome was a dummy indicator for the SNF selected among the set of available SNFs at discharge, known as the choice set. Consistent with previous research, we assigned choice sets that were the same for all patients being discharged from the same hospital regardless of ADRD status.^25,26^ For each patient, the choice set is equal to the union of all SNFs in a 24-km radius of the discharging hospital, the nearest 15 SNFs to the hospital, and all SNFs identified in the hospital’s Medicare claims. The number of records in our data set equaled the number of patients times the number of SNFs in each patient’s choice set. Our model predicts for each patient the probability of discharge to all potential SNFs, the sum of which equals 1.

The main explanatory variable in the conditional logit analysis is SNF quality. We included other SNF attributes as covariates, including bed size, occupancy rate, share of Medicare financing, and therapy staff level (reflecting postacute care specialization), for-profit ownership, hospital-based status, presence of a dementia special care unit, exact euclidean distance from discharging hospital to potential SNFs, and distance from the zip code tabulation area centroid to potential SNFs. Critically, the distance variables capture neighborhood and market effects or potential availability-based differences in SNF quality that could arise due to ADRD-related differences in neighborhood or hospital location.

The estimated conditional logit coefficients measure the independent association between each explanatory variable and SNF selection. We stratified models by ADRD status to examine whether the association between SNF quality and selection differed between patients with and without ADRD. We tested for differences more formally by including an interaction term between ADRD status and all explanatory variables in a specification that included both patient subpopulations. A weaker association between SNF quality and selection among patients with ADRD, along with a significant interaction term, provides evidence for disparity in SNF quality for those with ADRD relative to those without ADRD. To facilitate the interpretability of the conditional logit model estimates, we converted coefficients to the adjusted probability of entering an SNF after simulating its star rating to be low, average, or high and assuming, for estimation purposes, patients enter the SNF nearest to their residence. We discuss how we derived these estimates from the conditional logit model in the eMethods in Supplement 1.

We conducted 2 main sensitivity analyses. First, we estimated the conditional logit model using the overall star rating of the SNFs as the main explanatory variable (instead of staffing) because the star rating is more prominently featured on Care Compare and may play a larger role in the discharge process. Second, we conducted several subgroup analyses. Individual fixed effects are included in a conditional logit analysis, but there may be differences in SNF quality related to certain patient characteristics, including race and ethnicity, that are more or less prevalent in the ADRD or non-ADRD subpopulation. For example, dual Medicaid enrollment is associated with lower SNF quality and patients with ADRD are more likely to be dually enrolled.^26^ While such patient characteristics cannot be controlled for directly in this approach, their impact can be assessed through subgroup analyses. The following subgroups were examined: dual Medicaid enrollees and Medicare-only patients; Black and White patients (binary classification was made owing to small number of participants in other racial and ethnic minority groups); patients who had surgery; and patients with high postacute care volume conditions (eg, heart failure, chronic obstructive pulmonary disease, pneumonia, and septicemia) whose SNF destination may vary less by ADRD status due to having more typical rehabilitation needs.

Data were analyzed with Stata MP, version 16.0 (StataCorp LLC). Null hypotheses were tested assuming a 2-sided type I error probability of .05. Robust SEs were used in all analyses. Analyses excluded observations with any missing data.

## Results

The full sample included 2 619 464 patients (22% with ADRD); mean (SD) age was 81.3 (8.6) years, 61% of the patients were women, 39% were men, 8% were Black, 87% were White, and 5% were of other race or ethnicity (ie, American Indian or Alaska Native, Asian or Pacific Islander, Hispanic, unknown, and other) (Table 1). Patients with ADRD were more frequently admitted to larger SNFs with a mean (SD) bed size of 129.0 (75.7) compared with 122.7 (73.9) for patients without ADRD. Patients with ADRD were more likely to enter for-profit SNFs (71% vs 67%), were less likely to enter hospital-based facilities (3% vs 6%), and had lower postacute care levels as indicated by the level of Medicare financing (mean [SD], 22.5 [18.1] vs 25.7 [20.8] share of beds). Patients with ADRD were more likely to enter SNFs with low star ratings (23% vs 20%) and less likely to enter SNFs with high star ratings (47% vs 53%).

**Table 1.** Individual, Residential, and Skilled Nursing Facility Characteristics of Patients With and Without Dementia

Individual or residential characteristic	No. (%)		
	All patients	ADRD	
		Yes	No
No.	2 619 464	585 674	2 033 790
Age, mean (SD)	81.3 (8.6)	84.9 (7.5)	80.3 (8.6)
Sex			
Male	1 008 825 (39)	222 865 (38)	785 960 (39)
Female	1 610 639 (61)	362 809 (62)	1 247 830 (61)
Race and ethnicity			
Black	219 189 (8)	56 166 (10)	163 023 (8)
White	2 269 627 (87)	499 117 (85)	1 770 510 (87)
Other^a^	130 648 (5)	30 391 (5)	100 257 (5)
Medicare-Medicaid dual enrollee	368 732 (14)	102 364 (17)	266 368 (13)
Elixhauser index, mean (SD)	9.2 (10.5)	9.7 (9.8)	9.1 (10.7)
Surgical DRG	976 065 (37)	137 687 (24)	838 378 (41)
Admission to emergency department	2 044 456 (78)	527 639 (90)	1 516 817 (75)
Hospital LOS, mean (SD)	8 (6.6)	7.6 (7.5)	8 (6.3)
Admission to ICU	829 974 (32)	166 401 (28)	663 573 (33)
Census region			
Northeast	577 207 (22)	125 976 (22)	451 231 (22)
Midwest	627 082 (24)	125 765 (21)	501 317 (25)
South	964 676 (37)	237 077 (40)	727 599 (36)
West	450 499 (17)	96 856 (17)	353 643 (17)
Rural county	437 137 (17)	87 926 (15)	349 211 (17)
Social Deprivation Index percentile, mean (SD)	45.9 (27.6)	46.2 (28)	45.9 (27.5)
Skilled nursing facility characteristic			
Total beds, mean (SD)	124.1 (74.3)	129.0 (75.7)	122.7 (73.9)
Occupancy rate, mean (SD)	81.9 (14.9)	82.4 (14.3)	81.7 (15)
For profit	1 781 820 (68)	414 629 (71)	1 367 191 (67)
Hospital-based	135 608 (5)	19 481 (3)	116 127 (6)
% Medicare-financed residents, mean (SD)	25 (20.2)	22.5 (18.1)	25.7 (20.8)
Contains dementia care unit	338 884 (13)	80 589 (14)	258 295 (13)
Staffing star rating			
Low (1-2)	524 946 (20)	133 399 (23)	391 547 (20)
Average (3)	720 139 (28)	170 216 (29)	549 923 (28)
High (4-5)	1 331 548 (52)	273 709 (47)	1 057 839 (53)

Abbreviations: ADRD, Alzheimer disease and related dementias; DRG, diagnosis-related group; ICU, intensive care unit; LOS, length of stay.

^a^ This category contains individuals of the following racial and ethnic groups, per the Medicare enrollment file: Asian, Hispanic, and North American Native, other, and unknown. Binary classification of Black and White race and ethnicity was made owing to small number of participants in other racial and ethnic minority groups.

**Figure 1.**
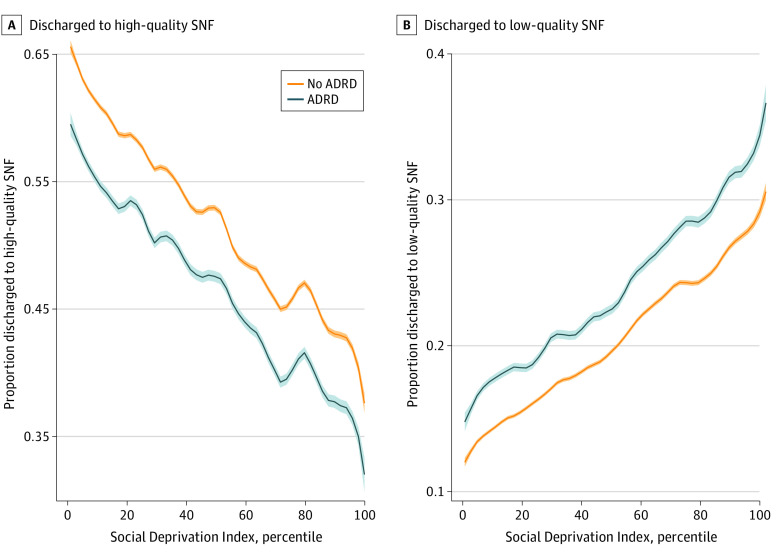
Differences in Skilled Nursing Facility (SNF) Quality Across Neighborhoods by Alzheimer Disease and Related Dementias (ADRD) Status High-quality SNFs are those with 4- or 5-star staffing ratings (scored 1 to 5). Low-quality SNFs are those with 1- or 2-star staffing ratings. Smooth curves and 95% CIs were estimated using local polynomial regression. Solid lines indicate the estimated probability of entry to low- and high-quality SNFs at each Social Deprivation Index level; shaded areas, 95% CIs.

Figure 1 shows the proportion of patients with and without ADRD admitted to low- and high-quality SNFs by neighborhood SDI level. Across neighborhoods, patients with ADRD were consistently admitted at higher rates to low-quality SNFs and at lower rates to high-quality SNFs compared with patients without ADRD. For example, among patients residing in the most-deprived neighborhoods (highest SDI), the rate of high-quality SNF entry was 31% for patients with ADRD compared with 35% for those without ADRD. Among patients in the least-deprived neighborhoods, the rate of high-quality SNF entry was 60% for patients with ADRD compared with 66% for those without ADRD.

Table 2 lists the adjusted association between SNF quality and other SNF attributes and SNF selection at discharge for patients with and without ADRD. There was a stronger association between SNF quality and the SNF selected at discharge for patients without ADRD compared with patients with ADRD. The log-odds of being discharged to an SNF with an average star rating was significantly higher than being discharged to an SNF with a low star rating, by 0.23 for patients with ADRD and by 0.29 for those without ADRD (difference, −0.06; *P* < .001). The log-odds of being discharged to an SNF with a high star rating was also significantly higher than being discharged to an SNF with a low star rating, by 0.31 for patients with ADRD and by 0.47 SNF for those without ADRD (difference, −0.16; *P* < .001). Thus, while a higher star rating was associated with an increased likelihood of SNF selection for both patients with and without ADRD, the association was stronger for patients without ADRD, as indicated by the statistical significance of the difference terms (coefficients on the interaction between ADRD status and star rating). What this means is that, holding other SNF attributes constant, patients with ADRD were less likely to be admitted to higher quality SNFs, especially those with high star ratings of 4 or 5.

**Table 2.** Adjusted Association Between SNF Attributes and SNF Choice for Patients With and Without Dementia^a^

Covariate	ADRD				Difference	
	Yes		No			
	Log-odds coefficient (SE)	*P* value	Log-odds coefficient (SE)	*P* value	Log-odds coefficient (SE)	*P* value
Staffing star rating						
Low (1-2)	1 [Reference]		1 [Reference]		1 [Reference]	
Average (3)	0.2323 (0.0098)	<.001	0.2897 (0.0080)	<.001	−0.0574 (0.0126)	<.001
High (4-5)	0.3143 (0.0102)	<.001	0.4721 (0.0082)	<.001	−0.1578 (0.0131)	<.001
Distance from discharging hospital to SNF	−0.5018 (0.0024)	<.001	−0.4853 (0.0019)	<.001	−0.0165 (0.0031)	<.001
Distance from residence to SNF	−1.4349 (0.0040)	<.001	−1.5507 (0.0031)	<.001	0.1159 (0.0051)	<.001
Total beds	0.0035 (0.0000)	<.001	0.0040 (0.0000)	<.001	−0.0005 (0.0001)	<.001
Occupancy rate	1.0937 (0.0268)	<.001	1.1993 (0.0201)	<.001	−0.1056 (0.0335)	.002
For-profit	−0.0649 (0.0082)	<.001	−0.1262 (0.0063)	<.001	0.0613 (0.0103)	<.001
% Medicare	0.0248 (0.0002)	<.001	0.0316 (0.0001)	<.001	−0.0068 (0.0002)	<.001
Hospital-based	−1.0288 (0.0238)	<.001	−0.5079 (0.0147)	<.001	−0.5209 (0.0279)	<.001
FTEs						
Physical therapist	−0.0002 (0.0011)	.88	0.0037 (0.0008)	<.001	−0.0038 (0.0013)	.002
Occupational therapist	0.0066 (0.0018)	<.001	0.0126 (0.0007)	<.001	−0.0060 (0.0019)	.004
Speech pathologist	−0.0005 (0.0004)	.26	−0.0034 (0.0018)	.06	0.0028 (0.0019)	.12
Contains dementia care unit	0.1861 (0.0104)	<.001	0.0282 (0.0083)	<.001	0.1578 (0.0133)	<.001

Abbreviations: ADRD, Alzheimer disease and related dementias; FTEs, full-time equivalents; SNF, skilled nursing facility.

^a^ Because the conditional logit estimation procedure is highly computationally intensive, the conditional logit was run on a random 10% sample of patients without ADRD and 20% sample of patients with ADRD. A larger share of the ADRD subpopulation was sampled to achieve a more comparable sample size for the estimation. The decimal places reflect that some estimates are small due to the scale of the explanatory variable.

There were several other SNF attributes associated with SNF selection that differed according to ADRD status. For instance, the log-odds of selecting an SNF significantly decreased if it was for-profit by −0.07 for patients with ADRD and by −0.13 for patients without ADRD (difference, 0.06; *P* < .001). Thus, despite the negative association of for-profit status with SNF selection for both populations, patients with ADRD were more likely than those without ADRD to be admitted to a for-profit SNF. Some SNF attributes were associated with SNF selection for only 1 of the patient subpopulations. For example, an SNF having a dementia special care unit increased the log-odds of selection only among patients with ADRD.

**Figure 2.**
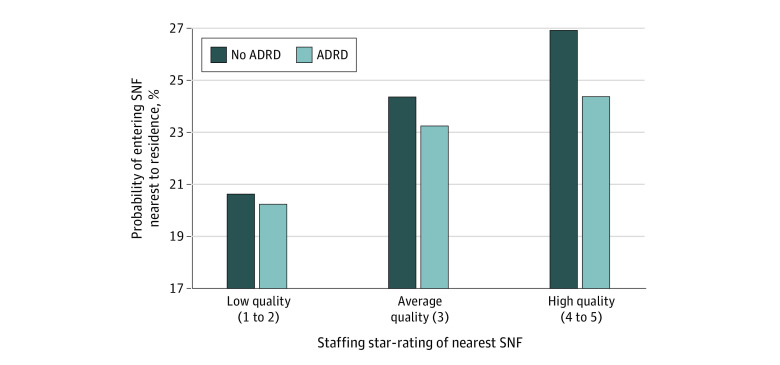
Adjusted Mean Probability of Entering the Nearest Skilled Nursing Facility (SNF) Based on Staffing Star Rating The method for deriving adjusted mean estimates from the conditional logit model is described in the eMethods in Supplement 1. ADRD indicates Alzheimer disease and related dementias.

Figure 2 shows the adjusted mean probability of entering the SNF nearest to a patient’s residence after simulating its star rating to be low, average, or high. If the nearest SNF was assigned a low star rating, the probability of entering it was 20.2% for patients with and 20.6% for those without ADRD. When the nearest SNF star rating was average, the probability of entering it was 23.2% for patients with ADRD and 24.4% for patients without ADRD. When the nearest SNF star rating was high, the probability of entering it was 24.4% for patients with ADRD and 26.9% for patients without ADRD. Thus, the difference in admission rate between patients with and without ADRD increased in accordance with SNF quality level.

The finding that patients with ADRD were significantly less likely than those without ADRD to enter higher quality SNFs was consistent in a sensitivity analysis that used the overall star rating rather than the staffing star rating (eTable 2 in Supplement 1). Furthermore, in subgroup analyses, we found that patients with ADRD were less likely to enter high-quality SNFs among dual Medicaid and nondual enrollees, Black patients, White patients, patients with a high-volume condition, and those who underwent surgery (eTable 3 in Supplement 1).

## Discussion

We found a disparity in the quality of postacute care received at nursing homes between individuals with and without ADRD. After accounting for differences in hospital, residential neighborhood, and different attributes of all potential SNF destinations, patients with ADRD were significantly less likely to be discharged to higher quality facilities. These findings are particularly striking because we excluded long-stay nursing home residents, who are frequently hospitalized and typically originate from lower-resourced facilities predominantly financed by Medicaid.^9,17,18^ Even if patients with ADRD had long-term care needs, nursing homes initially received compensation from Medicare for providing postacute care to the entirety of the study population. Results were consistent in subgroup analyses that examined ADRD-based disparities within racial and ethnic groups among patients with common postacute care diagnoses.

Persons with ADRD may be discharged to lower-quality postacute nursing home care for several reasons. For instance, as noted in qualitative reports, SNF managers may believe Medicare reimbursement inadequately considers patients’ behavioral health.^27^ The Resource Utilization Groups payment system in place during this study’s period rewarded therapy provision and did not consider the behavioral complexity that characterizes the ADRD population. Since patients with ADRD may be less likely to participate in the maximum hours of therapy, this would necessarily limit Medicare reimbursement rates. Even the new Patient Driven Payment model, which places greater emphasis on patients’ needs for skilled nursing, has not increased payment for behavioral issues.^28^

Incentives tied to postacute care length of stay may also be associated with disparities. For example, nursing homes are increasingly held accountable for whether patients receiving postacute care are successfully discharged to the community.^29^ Furthermore, if a patient cannot be discharged, the nursing home faces a potentially large decrease in revenue because an SNF benefit period lasts for 100 days at maximum. After 100 days, traditional Medicare beneficiaries pay for postacute care out-of-pocket or through Medicaid, which generally reimburses at a fraction of the Medicare rate. Out-of-pocket payers lacking indefinite funds eventually spend down their incomes to qualify for Medicaid financing. Thus, if patients with ADRD have a lower likelihood of leaving the nursing home after their postacute care needs are met, then nursing homes may avoid admitting them to prevent possible future shortfalls in revenue. Selectivity in admissions in the nursing home industry has been demonstrated empirically and may be more frequent among higher quality facilities given greater demand for their services.^27,30^

Public reporting initiatives may also affect differences in nursing home quality between patients with and without ADRD. A study by Konetzka and colleagues^10^ reported that nursing homes improved their quality over time following the release of star ratings in 2009, but improvement was lower for nursing homes disproportionately serving Medicare-Medicaid dual enrollees. A suggested reason for the disparity after public reporting was initiated is inadequate access to web-based information. For patients with ADRD, cognitive impairment affects computer literacy and thus the ability to use web-based ratings.^11^

The adverse association of quality improvement initiatives with quality-of-care disparities is a common feature of many Medicare programs.^31,32,33^ Improving nursing home quality for patients with ADRD likely requires more targeted efforts and is essential given research showing improved outcomes for individuals discharged to higher rated SNFs.^34^ Some studies also have found that residents of higher rated nursing homes have had better outcomes during the COVID-19 pandemic.^35,36^ Addressing postacute care reimbursement mechanisms is a potential avenue, given considerable research linking improvements in nursing home quality with increases in long-term care reimbursement rates.^37,38^ Broadly, however, policies that recognize the interrelationship between postacute and long-term care and specifically address perverse incentives stemming from fragmentation are likely needed to ensure that patients with ADRD receive equitable postacute and long-term care.

### Limitations

This study has limitations. First, our findings may not be generalizable to certain populations, such as persons younger than 65 years, Medicare Advantage enrollees, and individuals who had recently resided within a nursing home prior to hospitalization. Second, the prevalence of ADRD in our study is likely underestimated because ascertainment was based on in-hospital billing codes. Historically, the recognition, diagnosis, and documentation of cognitive impairment during acute care management is known to be insufficient. Third, our study used star ratings to approximate SNF quality, which some research suggests are best suited for capturing quality at the extremes.^39^ Star ratings are also, like many other publicly reported quality measures, derived from data submitted by SNFs. Fourth, our conditional logit model only included SNF characteristics observed in administrative data sources, but there may be unobserved attributes associated with SNF quality and ADRD status that may affect our estimates of disparity. For example, an SNF that has a stronger interorganizational tie to the discharging hospital may receive more lower-risk referrals.^40^ Fifth, our study population only included patients discharged to SNFs. The availability and quality of other postacute care options, such as home health agencies, is not accounted for in our analysis and may be related to the likelihood of discharge to a low-quality SNF. For example, in less-competitive or less-saturated markets with fewer alternatives to SNF care, disparities in SNF quality for persons with ADRD may increase. Sixth, our analysis did not identify the cause of lower SNF quality for patients with ADRD, as our statistical model cannot distinguish between the role of patients, hospitals, SNFs, and other actors in the SNF selection process. While our approach accounted for hospital, neighborhood, and patient characteristics known to be associated with SNF quality in subgroup analyses, the contribution of patients (demand-side factors) vs other actors (supply-side factors) to the ADRD-based disparity in SNF quality remains uncertain. For many policy purposes, however, knowledge of the unequal allocation of SNF quality between patients with and without ADRD is highly relevant.

## Conclusions

In this cross-sectional study, patients with ADRD, who are disproportionately more likely to require postacute care after hospitalization, had a greater likelihood to be discharged to lower-quality SNFs after accounting for differences in hospital, residential neighborhood, and the different characteristics of the set of SNFs available at discharge. Enhancing incentives to provide postacute care to patients with ADRD may be necessary to improve access to better facilities, which may ultimately reduce the importance of ADRD as a marker of disparity.

## Notes

Supplement 1. **eMethods.** Detailed Methods **eTable 1.** Study Data Sources **eTable 2.** Association Between Overall Star Rating and Skilled Nursing Facility Choice for Patients With and Without Dementia **eTable 3.** Subgroup Analyses of the Association Between Skilled Nursing Facility Quality and Skilled Nursing Facility Choice for Patients With and Without Dementia
eReferences

Supplement 2.
Data Sharing Statement
