# Supplementary material for: Risk of Discharge to Lower-Quality Nursing Homes Among Hospitalized Older Adults With Alzheimer Disease and Related Dementias
Source: JAMA Netw Open. 2023 Feb 8;6(2):e2255134. doi: 10.1001/jamanetworkopen.2022.55134 (PMC9909503; doi:10.1001/jamanetworkopen.2022.55134)
Supplement: Supplement 1. — eMethods. Detailed Methods eTable 1. Study Data Sources eTable 2. Association Between Overall Star Rating and Skilled Nursing Facility Choice for Patients With and Without Dementia eTable 3. Subgroup Analyses of the Association Between Skilled Nursing Facility Quality and Skilled Nursing Facility Choice for Patients With and Without Dementia eReferences [file jamanetwopen-e2255134-s001.pdf]

Supplementary Online Content

Kosar CM, Mor V, Werner RM, Rahman M. Risk of discharge to lower-quality nursing homes among hospitalized older adults with Alzheimer disease and related dementias. *JAMA Netw Open*. 2023;6(2):e2255134. doi:10.1001/jamanetworkopen.2022.55134

eMethods. Detailed Methods

eTable 1. Study Data Sources

eTable 2. Association Between Overall Star Rating and Skilled Nursing Facility Choice for Patients With and Without Dementia

eTable 3. Subgroup Analyses of the Association Between Skilled Nursing Facility Quality and Skilled Nursing Facility Choice for Patients With and Without Dementia

eReferences

This supplementary material has been provided by the authors to give readers additional information about their work.

© 2023 Kosar CM et al. *JAMA Network Open.*

eMethods. Detailed Methods

Data Sources

This study relies on individual-, health care facility-, and community-level data sources. Individual-level data were obtained from the Medicare Beneficiary Summary File (Medicare enrollment data), the Medicare Provider and Analysis Review claims files (MedPAR), and Minimum Data Set (MDS) nursing home resident assessments. Facility-level data were obtained from the Certification and Survey Provider Enhanced Reports database (nursing home organizational characteristics), Medicare's Care Compare website (nursing home quality), and American Hospital Association annual surveys (hospital characteristics). The Social Deprivation Index (SDI), a ZIP code tabulation area (ZCTA) measure, was used to characterize patients' residences.[1] The SDI is a composite measure of economic disadvantage based on socioeconomic measures collected in the American Community Survey and ranges from 1 (least disadvantaged) to 100 (most disadvantaged). All data sources are listed and described in detail in **eTable 1**.

Sample

We combined enrollment data with hospital and SNF claims to identify hospitalized Traditional Medicare beneficiaries who were discharged directly to a SNF from 2017 through 2019. We applied some exclusion criteria. First, we excluded Medicare Advantage enrollees because MedPAR does not include SNF claims for these individuals. Second, to reduce heterogeneity, we excluded individuals aged below 65 years. Third, we excluded those with a nursing home stay within one year of their index hospitalization, identified through both claims and MDS data. This exclusion serves multiple purposes: it removes long-stay nursing home residents who are frequently cycled in and out of hospitals[2-3]; it ensures patients were treated under a new SNF benefit period; and it removes individuals who previously selected a SNF for post-acute care, which is important for analyses of discharge location.

Dementia Diagnosis

We classified patients as having ADRD based on the presence of an International Classification of Diseases tenth revision (ICD-10) code indicating dementia in any of the 25 discharge diagnosis fields of the hospital claim. With this approach ADRD status is assigned prior to SNF admission, which is important for analyzing disparities in future discharge location. ICD-10 codes for ADRD were obtained from Medicare's Chronic Condition Warehouse algorithm.[4]

Skilled Nursing Facility Quality

SNF quality was quantified with existing 5-star ratings reported on Care Compare. Although there are a variety of quality measures available on Care Compare, star-ratings are the most visible to patients, families, and discharge planners and are thus well-suited for analyses of discharge location. SNFs are assigned ratings on three primary domains: staffing, health inspections, and quality measures (e.g., past performance on patient outcomes) which are then summarized into an overall rating. We focus on the staffing rating for three reasons. First, staffing has been consistently linked to better outcomes for nursing home patients.[5-8] Second, while quality of care is multifaceted and intangible in many ways, it is difficult to imagine the provision of high-quality care in an institutional setting without a sufficient number of and adequately trained clinical staff. Third, the factors determinative of the staffing-rating, which today include direct care staffing levels and turnover rates, may be more easily modifiable or subject to regulation compared with the factors determinative of the other star-ratings. We grouped SNFs by three categories of staffing star-rating: 1-2 stars [reference group], 3 stars, and 4-5 stars. This facilitates statistical modeling as well as interpretation since the classifications intuitively correspond to 'low', 'medium', and 'high' SNF quality.

Statistical Analysis

We first show the average characteristics of patients with and without ADRD including the quality level and other attributes of their destination SNFs. Patients are known to prefer SNFs nearer to their residences, and neighborhood income levels are known to be associated with SNF quality.[9-12] Because we wanted to examine the relationship between ADRD and SNF quality independent of neighborhood, we also examined this relationship stratified by SDI level.

We then formally tested for differences in the likelihood of entering higher quality SNFs between patients with and without ADRD using a conditional logit (discrete choice) model.[13-14] This approach has the advantage of being able to simultaneously account for numerous SNF characteristics across the set of SNFs a patient is choosing between. The real discharge process involves consideration of more than one SNF, the proximity of these SNFs, quality, and other SNF characteristics. Some SNF characteristics may be strongly correlated (e.g., location and

© 2023 Kosar CM et al. *JAMA Network Open.*

quality) or matter more depending on ADRD status. For example, patients with ADRD or their proxies may find a SNF with a dementia special care unit (SCU) to be more valuable than a SNF with a higher star-rating. Not accounting for these issues may mask larger differences in SNF quality by ADRD status. The conditional logit approach, however, models the observable attributes of all competing SNFs simultaneously.

In our analysis the outcome was a dummy indicator for the SNF selected among the set of available SNFs at discharge, known as the "choice set." Consistent with previous research, we assigned choice sets that were the same for all patients being discharged from the same hospital regardless of ADRD status.[15-16] For each patient the choice set is equal to the union of all SNFs in a 15-mile radius of the discharging hospital, the nearest 15 SNFs to the hospital, and all SNFs the hospital has ever discharged patients to. Altogether, the criteria for specifying the choice set yields a realistic set of options for patients regardless of the population density of the area surrounding the hospital or their residences. For example, the nearest SNF criterion alone would yield choice sets smaller than what would be expected for patients hospitalized in urban areas. By contrast, the 15-mile radius criterion would yield choice sets smaller than what would be expected for patients hospitalized in rural areas.

The main explanatory variable in the conditional logit analysis is SNF quality. We included other SNF attributes as covariates, including: bed size, occupancy rate, share of Medicare financing and therapy staff level (reflecting post-acute care specialization), for-profit ownership, hospital-based status, presence of a dementia SCU, exact Euclidean distance from discharging hospital to potential SNFs, and distance from a patient's ZCTA centroid to potential SNFs. The distance variables capture preferences for receiving treatment at a more nearby SNF, which may compete with preferences for higher quality SNFs. More broadly, however, these distance measures capture neighborhood and market effects, or potential availability-based differences in SNF quality that could arise due to ADRD-related differences in neighborhood or hospital location. Another important distance-based preference is nearness to an informal caregiver, particularly for patients with ADRD. Although this may be correlated with the distance to a patient's ZCTA, it cannot be derived from our data sources and thus was not included in our analysis.

The estimated conditional logit coefficients measure the independent association between each explanatory variable and SNF selection. We stratified models by ADRD status to see how the association between SNF quality and selection differed between patients with and without ADRD. We tested for differences more formally by including an interaction term between ADRD status and all explanatory variables in a specification that included both patient subpopulations. A weaker association between SNF quality and selection among patients with ADRD, along with a significant interaction term, provides evidence for disparity in SNF quality for those with ADRD relative to those without ADRD. To facilitate the interpretability of the conditional logit model estimates, we convert coefficients to the adjusted mean probability of entering a low-, average-, or high-quality nursing SNF for patients with and without ADRD.

Calculation of Adjusted Mean Predictions.

Because the preference coefficients estimated from a conditional logit model are difficult to apply practically, we translate these results to adjusted mean probabilities. However, adjusted mean probabilities derived from a conditional logit model predicting (SNF) choice among a set of alternatives are alternative-specific estimates which cannot be calculated using conventional techniques. As such, we chose to calculate the probability patients entered the SNF nearest to their residences (the nearest alternative) after simulating it to have a 'low', 'average', or 'high' star-rating. To do this, we first estimate the conditional logit model and obtain model parameters derived from the observed data. We estimate the model separately for patients with and without ADRD to allow parameter estimates to vary by ADRD status. We then assign the nearest SNF in patient's choice set to a low (1-2) star-rating, regardless of whether the SNF was chosen or not or what the observed star-rating was. We then, using the original model parameters, estimate the probability of entering the nearest SNF given the low-star rating and pool predictions across patients (by ADRD status). We repeat this process for average and high-star rating. The resulting adjusted mean predictions, depicted in **Figure 2**, can be interpreted as the probability of entering the nearest SNF if its star-rating was changed to low, average, or high, holding the SNFs other characteristics and the characteristics of all competing facilities constant.

Sensitivity Analyses

We conducted two main sensitivity analyses. First, we estimated the conditional logit model using SNFs' overall star-rating as the main explanatory variable (instead of staffing) because it is more prominently featured on Care Compare and may have a stronger role in the discharge process. Second, we conduct several subgroup analyses. Individual fixed effects are effectively included in a conditional logit analysis, but there may be differences in SNF quality related to certain patient attributes that are more or less prevalent in the ADRD or non-ADRD subpopulation. For example, dual Medicaid enrollment is associated with lower SNF quality and patients with ADRD are more

© 2023 Kosar CM et al. *JAMA Network Open.*

likely to be dually enrolled.[16] While such patient characteristics cannot be controlled for directly in this approach, their impact can be assessed through subgroup analyses. The following subgroups were examined: dual Medicaid enrollees and Medicare-only patients; Black and White patients (other racial/ethnic groups were not examined due to low prevalence); and patients with high post-acute care volume conditions (e.g., heart failure, chronic obstructive pulmonary disease, pneumonia, and septicemia) who's SNF destination may vary less by ADRD status due to having more typical rehabilitation needs.

Data were analyzed with Stata MP 16.0 (StataCorp). Null hypotheses were tested assuming a two-sided type I error probability of 0.05. Robust standard errors were used in all analyses.

© 2023 Kosar CM et al. *JAMA Network Open.*

eTable 1. Study Data Sources

Data Source	Data Unit	Description
Medicare Beneficiary Summary File (MBSF)	Individual	Includes for all currently or previously enrolled Medicare beneficiaries, data on demographics, payer source (e.g., Medicare Advantage or dual Medicaid enrollment), entitlement reason, residential zip code, and date of death
Medicare Provider and Analysis Review (MedPAR) files	Individual	Include 100% of claims related to acute and organized post-acute care for Traditional Medicare beneficiaries. The inpatient and SNF claims contain dates of care, types of services, diagnosis codes, and reimbursements
Minimum Data Set (MDS)	Individual	A clinical assessment tool administered to all individuals admitted to government-certified nursing homes. Patients/residents are assessed upon facility admission and periodically thereafter until the final discharge assessment. In this study MDS assessments were used to identify prior nursing home use.
Certification and Survey Provider Enhanced Reports (CASPER)	Nursing home	Annual nursing home surveys that contain facility organizational characteristics (e.g. facility size, proprietary status, staffing levels) and some aggregate data on residents such as payer mix.
Care Compare report cards	Nursing home	The Medicare Care Compare website (https://www.medicare.gov/care-compare/) provides consumers with report cards that list several types of data pertaining to nursing home quality, including user-friendly 5 star-ratings. Each facility receives an overall star-rating, as well as ratings for specific domains such as health inspections and staffing.
American Hospital Association (AHA) annual survey	Hospital	Annual hospital surveys that contain facility organizational characteristics. In this study we used the AHA data to obtain the geographic coordinates of all hospitals in our sample
Social Deprivation Index (SDI)	Zip Code Tabulation Area	The SDI was developed by the Robert Graham Center and is a composite measure of economic disadvantage that ranges from 1 (least deprived) to 100 (most deprived). Published SDI scores are constructed from a factor analysis of seven socioeconomic indicators recorded in the American Community Survey. The seven socioeconomic indicators are: the poverty rate, the percentage of adults with less than a high school-level education, the percentage of households with a single parent head, the percentage of the population living in rental units, the percentage of the population living in overcrowded housing, the percentage of households without a car, and the unemployment rate.

© 2023 Kosar CM et al. *JAMA Network Open.*

eTable 2. Association between Overall Star Rating and Skilled Nursing Facility Choice for Patients with and without Dementia

	ADRD	No ADRD	Difference
Covariate	Coef. (SE)	Coef. (SE)	Coef. (SE)
3 Star Overall rating (Ref. 1-2)	0.2358*** (0.0106)	0.2692*** (0.0086)	-0.0334* (0.0137)
4-5 Star Overall rating (Ref. 1-2)	0.3617*** (0.0089)	0.4894*** (0.0071)	-0.1277*** (0.0113)
Distance from discharging hospital to SNF	-0.5065*** (0.0025)	-0.4916*** (0.0019)	-0.0149*** (0.0031)
Distance from residence to SNF	-1.4347*** (0.0040)	-1.5497*** (0.0031)	0.1150*** (0.0051)
Total beds	0.0036*** (0.0000)	0.0041*** (0.0000)	-0.0005*** (0.0000)
Occupancy rate	0.9797*** (0.0266)	1.0595*** (0.0200)	-0.0798* (0.0333)
For profit	-0.0564*** (0.0081)	-0.1295*** (0.0061)	0.0730*** (0.0101)
% Medicare	0.0241*** (0.0002)	0.0308*** (0.0001)	-0.0067*** (0.0002)
Hospital-based	-1.0205*** (0.0236)	-0.4935*** (0.0146)	-0.5270*** (0.0278)
Physical therapist FTEs	-0.0008 (0.0012)	0.0014 (0.0008)	-0.0021 (0.0014)
Occupational therapist FTEs	0.0045* (0.0019)	0.0112*** (0.0008)	-0.0067** (0.0020)
Speech pathologist FTEs	-0.0004 (0.0004)	-0.0029 (0.0017)	0.0025 (0.0017)
Contains dementia care unit	0.1914*** (0.0104)	0.0333*** (0.0083)	0.1581*** (0.0132)

Notes: *p<0.05, **p<0.01, ***p<0.001. ADRD=Alzheimer's and Related Dementias; SNF=Skilled Nursing Facility; Ref.=Reference; FTE=Full time equivalent. Because the conditional logit estimation procedure is highly computationally intensive, we ran the conditional logit on a random 10% sample of patients without ADRD and 20% sample of patients with ADRD. A larger share of the ADRD subpopulation was sampled to achieve a more comparable sample size for the estimation.

© 2023 Kosar CM et al. *JAMA Network Open.*

eTable 3. Subgroup Analyses of the Association between Skilled Nursing Facility Quality and Skilled Nursing Facility Choice for Patients with and without Dementia

	High-quality SNF		
	**No Dementia**	**Dementia**	**Difference**
**Characteristic**	**Coef. (SE)**	**Coef. (SE)**	**Coef. (SE)**
Medicare only	0.5490*** (0.0089)	0.3903*** (0.0089)	-0.1587*** (0.0126)
Dual Medicaid recipient	0.0763*** (0.0094)	-0.0331** (0.0105)	-0.1094*** (0.0141)
White	0.5040*** (0.0089)	0.3543*** (0.0088)	-0.1496*** (0.0125)
Black	0.2558*** (0.0084)	0.0709*** (0.0141)	-0.1849*** (0.0164)
Surgical DRG	0.5913*** (0.0107)	0.4455*** [0.0097]	-0.1458*** (0.0144)
Common PAC diagnosis	0.3883*** (0.0102)	0.3147*** (0.0108)	-0.0736*** (0.0149)
	**Average-quality SNF**		
	**No Dementia**	**Dementia**	**Difference**
**Characteristic**	**Coef. (SE)**	**Coef. (SE)**	**Coef. (SE)**
Medicare only	0.3365*** (0.0088)	0.2723*** (0.0086)	-0.0642*** (0.0123)
Dual Medicaid recipient	0.1239*** (0.0086)	0.0754*** (0.0095)	-0.0485*** (0.0128)
White	0.3172*** (0.0087)	0.2440*** (0.0084)	-0.0732*** (0.0121)
Black	0.2381*** (0.0077)	0.1636*** (0.0125)	-0.0744*** (0.0147)
Surgical DRG	0.3524*** (0.0106)	0.2874*** [0.0094]	-0.0650*** (0.0141)
Common PAC diagnosis	0.2713*** (0.0099)	0.2213*** (0.0104)	-0.0500*** (0.0143)

Notes: * p<0.05, **p<0.01, ***p<0.001. ADRD=Alzheimer's and Related Dementias; SNF=Skilled Nursing Facility; Ceoff.=log-odds coefficient; SE=Standard error; DRG=Diagnosis Related Group; PAC=Post-acute care. Patients are considered to have a common PAC diagnosis if their primary discharge diagnosis was one of the following: congestive heart failure, chronic obstructive pulmonary disease, pneumonia, or septicemia. High-quality SNFs are those with 4 and 5 star-ratings on staffing. Average-quality SNFs are those with a 3 star-rating on staffing. The log-odds coefficients are derived from a conditional logit model and represent the association of star-rating with facility choice where a low-rating (1 or 2) stars is the reference category. Covariates in the conditional logit model are listed in the methods section and are not displayed in this table that focuses on the parameter of interest in several subgroup analyses. Because conditional logit is computationally intensive, we estimated the model on random samples of patients within each subgroup. The proportion sampled varied by subgroup by the amount necessary to achieve a panel size of at least 100,000 observations.

© 2023 Kosar CM et al. *JAMA Network Open.*

eReferences

1. Butler DC, Petterson S, Phillips RL, Bazemore AW. Measures of social deprivation that predict health care access and need within a rational area of primary care service delivery. Health Serv Res. 2013;48(2pt1):539-59.

2. Mor V, Intrator O, Feng Z, Grabowski DC. The revolving door of rehospitalization from skilled nursing facilities. Health Aff (Millwood). 2010;29(1):57-64.

3. Intrator O, Grabowski DC, Zinn J, et al. Hospitalization of nursing home residents: the effects of states' Medicaid payment and bed-hold policies. Health Serv Res. 2007;42(4):1651-71.

4. Chronic Conditions Data Warehouse. Condition categories. Accessed June 2021. https://www2.ccwdata.org/web/guest/condition-categories

5. Konetzka RT, Stearns SC, Park J. The staffing–outcomes relationship in nursing homes. Health Serv Res. 2008;43(3):1025-42.

6. Lin H. Revisiting the relationship between nurse staffing and quality of care in nursing homes: An instrumental variables approach. J Health Econ. 2014;37:13-24.

7. Foster AD, Lee YS. Staffing subsidies and the quality of care in nursing homes. J Health Econ. 2015;41:133-47.

8. Antwi YA, Bowblis JR. The impact of nurse turnover on quality of care and mortality in nursing homes: Evidence from the great recession. Am J Health Econ. 2018;4(2):131-63.

9. Mor V, Zinn J, Angelelli J, Teno JM, Miller SC. Driven to tiers: socioeconomic and racial disparities in the quality of nursing home care. Milbank Q. 2004;82(2):227-56.

10. Pesis-Katz I, Phelps CE, Temkin-Greener H, Spector WD, Veazie P, Mukamel DB. Making difficult decisions: the role of quality of care in choosing a nursing home. Am J Public Health. 2013;103(5):e31-7.

11. Shugarman LR, Brown JA. Nursing home selection: How do consumers choose? Volume II: Findings from the website content review. https://aspe.hhs.gov/reports/nursing-home-selection-how-do-consumers-choose-volume-ii-findings-website-content-review-1. 2006. Accessed June 2022.

12. Sharma H, Perraillon MC, Werner RM, Grabowski DC, Konetzka RT. Medicaid and nursing home choice: Why do duals end up in low-quality facilities?. J Appl Gerontol. 2020;39(9):981-90.

13. McFadden D. Conditional Logit Analysis of Qualitative Choice Behavior. Frontiers in Econometrics. New York: Academic Press; 1974. p. 105-42.

14. McFadden D. Modeling the choice of residential location. In: Karlqvist A, Lundqvist L, Snickars F, Weibull J, editors. Spatial Interaction Theory and Planning Models. North-Holland, Amsterdam1978. p. 75–96.

15. Rahman M, Foster AD. Racial segregation and quality of care disparity in US nursing homes. J Health Econ. 2015;39:1-6.

16. Rahman M, Grabowski DC, Gozalo PL, Thomas KS, Mor V. Are dual eligibles admitted to poorer quality skilled nursing facilities?. Health Serv Res. 2014;49(3):798-817.

© 2023 Kosar CM et al. *JAMA Network Open.*
