# Supplementary material for: Risk of Discharge to Lower-Quality Nursing Homes Among Hospitalized Older Adults With Alzheimer Disease and Related Dementias
Source: JAMA Netw Open. 2023 Feb 8;6(2):e2255134. doi: 10.1001/jamanetworkopen.2022.55134 (PMC9909503; doi:10.1001/jamanetworkopen.2022.55134)
Supplement: Supplement 2. — Data Sharing Statement [file jamanetwopen-e2255134-s002.pdf]

Data Sharing Statement

Kosar. Risk of Discharge to Lower-Quality Nursing Homes Among Hospitalized Older Adults With Alzheimer Disease and Related Dementias. *JAMA Netw Open*. Published February 08, 2023. doi:10.1001/jamanetworkopen.2022.55134

Data
Data available: No

Additional Information
Explanation for why data not available: Individual-level Medicare claims data are not allowed to be shared publicly
